# A Fluorogenic Biosensor for Direct Detection of *Vibrio vulnificus*, a Climate Change Biomarker

**DOI:** 10.1002/mbo3.70287

**Published:** 2026-04-21

**Authors:** M. Nieves Aranda, Isabel Caballos, Alba López‐Palacios, Héctor Carmona‐Salido, Eva Sanjuan, Elena Aznar, Carmen Amaro, Ramón Martínez‐Máñez, Andy Hernández‐Montoto

**Affiliations:** ^1^ Instituto Interuniversitario de Investigación de Reconocimiento Molecular y Desarrollo Tecnológico (IDM) Universitat de València Valencia Spain; ^2^ Unidad Mixta de Investigación en Nanomedicina y Sensores, Instituto de Investigación Sanitaria La Fe (IISLAFE) Universitat Politècnica de València Valencia Spain; ^3^ CIBER de Bioingeniería Biomateriales y Nanomedicina, Instituto de Salud Carlos III Spain; ^4^ Instituto Universitario de Biotecnología y Biomedicina (BIOTECMED) Universitat de València, Burjassot Valencia Spain; ^5^ Unidad Mixta UPV‐CIPF de Investigación en Mecanismos de Enfermedades y Nanomedicina, Centro de Investigación Príncipe Felipe Universitat Politècnica de València Valencia Spain; ^6^ Departamento de Química Universitat Politècnica de València Valencia Spain

**Keywords:** climate change, human health, molecular gates, nanoporous anodic alumina, oligonucleotide, optical sensors, *Vibrio vulnificus*

## Abstract

*Vibrio vulnificus*, a marine pathogen and climate change biomarker, poses serious risks to human and animal health through seafood consumption and seawater exposure. Rapid detection methods are urgently needed for both vibriosis diagnosis and surveillance in warming coastal waters. We report a fluorogenic biosensor based on nanoporous anodic alumina loaded with rhodamine B and capped with an oligonucleotide probe targeting a unique sequence of the *vvhA* cytolysin gene, specific to *V. vulnificus*. In the presence of the target DNA, the probe is displaced, pores open, and the fluorophore is released, generating a measurable signal. The biosensor exhibited high sensitivity and selectivity across diverse matrices, including fish mucus and serum, human serum, sterilized brackish water, and—critically—unprocessed natural lake and seawater samples, without DNA extraction or amplification. Detection limits ranged between 10² and 5 × 10² CFU mL⁻¹, comparable in sensitivity to state‐of‐the‐art qPCR assays. The biosensor outperformed conventional approaches in speed, simplicity, and cost‐effectiveness, while maintaining accuracy. These findings underscore the potential of this platform for integrated One Health applications, bridging environmental monitoring with rapid diagnosis of vibriosis in humans and animals.

Preliminary results from this study were previously made available as a preprint in SSRN (DOI: https://ssrn.com/abstract=5032822).

## Graphical abstract

Nanoporous anodic alumina (S2) loaded with rhodamine B and capped with a specific oligonucleotide probe (O1) for the *vvhA* gene. In the presence of *V. vulnificus* genomic DNA, probe displacement opens the pores and releases the dye, generating a measurable fluorescent signal.

## 1 Introduction

Climate change is intensifying the spread of marine pathogens such as *Vibrio vulnificus*, which thrives in brackish water ecosystems across temperate, tropical, and subtropical regions (Baker‐Austin et al. 2013; Baker‐Austin and Oliver 2018; Fleischmann et al. 2022). The warming of ocean waters and the resultant decrease in salinity, direct outcomes of global warming, are causing an expansion in both the abundance and geographic distribution of *V. vulnificus*, moving into areas previously cooler (Baker‐Austin et al. 2013; Baker‐Austin and Oliver 2018; Fleischmann et al. 2022). This pathogen causes vibriosis in humans and fish, a condition that in humans ranges from gastroenteritis to deadly fulminant septicemia within 24–48 h of infection (Amaro et al. 2020; Ceccarelli et al. 2019). Human vibriosis transmission occurs through the consumption of raw or undercooked seafood and contact with seawater or diseased animals (zoonosis) (Oliver 2015; Dalsgaard et al. 1996; Veenstra et al. 1992). Primary sepsis caused by ingestion of this bacterium is considered the leading cause of mortality among foodborne bacterial pathogens (Baker‐Austin et al. 2009).

Multiple immunological and genetic methods have been developed for the detection and identification of this species (Kassim et al. 2011; Cantet et al. 2013; Cañigral et al. 2010; Harwood et al. 2004). However, the detection of *V. vulnificus* from environmental samples usually requires the prior collection of pure cultures, which considerably lengthens the detection process. Among different methods, qPCR and LAMP (loop‐mediated isothermal amplification) stand out because, being the most sensitive (limit of detection around 10^2^ bacteria mL^−1^) the previous step of obtaining pure cultures could in some cases be obviated. Nonetheless, the former requires technical skills that might influence the efficiency of reaction amplification and the quality of standard curves, which affects the accuracy of detection, and the latter is sensitive to the external environment and may cause false positives (Surasilp et al. 2011; Han et al. 2011; Yang et al. 2020). In addition, these methods are expensive and complex as they require prior DNA extraction performed by qualified personnel using specialized equipment. In summary, although qPCR and LAMP are sensitive methods, they still face challenges related to cost, complexity, and the risk of false positives.

At the same time, the advent of biosensor technology has shown considerable promise in pathogen detection, including *V. vulnificus* (Dicle and Karamese 2024; Fan et al. 2021; Pérez‐Roig et al. 2024; Xiao et al. 2021; Wang et al. 2021). Among the biosensors developed, several approaches have demonstrated high sensitivity and specificity, including a fluorogenic DNAzyme‐based biosensor (Fan et al. 2021), an impedance‐based sensor that using a functionalized oligonucleotide probe (Pérez‐Roig et al. 2024), a recombinase‐assisted amplification (RAA)‐CRISPR/Cas12a system (Xiao et al. 2021), and an RAA‐based lateral flow biosensor (Wang et al. 2021). Although the limits of detection of these systems are usually below that of conventional PCR, they are not below that of qPCR.

Another line of work that may lead to improved sensors is based on the use of nanomaterials combined with biomolecules to give bioorganic–inorganic hybrid systems (Pla et al. 2019). Among different nanomaterials, nanoporous anodic alumina (NAA) has become one of the most widely used for sensor development due to its exceptional qualities, such as easily tunable surfaces and high loading capacity. NAA becomes a powerful detection and diagnostic tool when loaded with reporters and capped with specific biomolecules to obtain gated nanomaterials (Aznar et al. 2016; Sancenón et al. 2015). This is generally carried out using alkoxysilane chemistry that allows different (bio)molecules to be easily attached to the outer surface of NAA (Baranowska et al. 2014; Llopis‐Lorente et al. 2017; Caballos et al. 2026). Following this approach, different nucleic acids, such as DNA, RNA, and aptamers, have been widely used as gatekeepers on mesoporous supports and have demonstrated outstanding applications (Ribes et al. 2018; Garrido‐Cano et al. 2021; Ribes et al. 2019). For instance, some works reported the use of DNA and mesoporous materials to develop gated‐nanodevices for the identification of different microorganisms, including viruses (Pla et al. 2021; López‐Palacios et al. 2025; Caballos et al. 2023; Hernández‐Montoto et al. 2023).

Based on the above, we report herein a gated nanostructured hybrid biosensor for the optical detection of *V. vulnificus*. The biosensor is based on the NAA scaffold that is loaded with Rhodamine B (RhoB) and capped with a specific oligonucleotide containing the base sequence of the *V. vulnificus* cytolysin structural gene (*vvhA*). The oligonucleotide inhibits fluorophore release by blocking the pores. In the presence of *V. vulnificus* DNA the capping oligonucleotide is displaced, allowing the pore opening and RhoB delivery. The sensitivity and specificity of the biosensor were evaluated with different types of laboratory‐contaminated natural samples (fish mucus and serum; human serum; and sterilized natural brackish water) and further assessed in natural lake and seawater samples. Remarkably, *V. vulnificus* could be successfully detected directly from natural water samples without the need for prior DNA extraction or amplification steps.

Consequently, this novel design enables direct and specific detection of *V. vulnificus*. The present work should be regarded as a proof‐of‐concept demonstration rather than a complete validation, but it represents a step forward toward environmental monitoring and diagnosis of vibriosis in humans and animals.

## 2 Material and Methods

### 2.1 Techniques

NAA surfaces were characterized by High Resolution Field Emission Scanning Electron Microscopy (HR‐FESEM). NAA systems were placed onto Al disks using carbon cement. Images were acquired using an HR‐Field Emission Scanning electron microscope (ZEISS GeminiSEM 500) operating at 5 kV. The atomic composition of analyzed surfaces was determined by Energy Dispersive X‐ray Spectroscopy (EDXS) using an X‐ray detector coupled to the microscope.

### 2.2 Chemicals

3‐aminopropyltriethoxysilane (APTES), hydrochloric acid, RhoB, magnesium chloride (anhydrous), acetonitrile (anhydrous), toluene (anhydrous), and tris(hydroxymethyl)aminomethane (TRIS) were acquired from Sigma‐Aldrich (Spain). InRedox Company (Longmont, CO, USA) provided NAA supports (Anodized aluminum oxide (AAO) film on Al substrate size 25 × 75 mm, AAO pore diameter 5 ± 2 nm, AAO thickness 10 ± 0.5 μm). Oligonucleotide (5′‐GAG CTG TCA CGG CAG TTG GAA CCA‐3′) (O1) and its complementary (5′‐TGG TTC CAA CTG CCG TGA CAG CTC‐3′) (O1c) were obtained from Thermo Fisher Scientific (Madrid, Spain).

### 2.3 Synthesis of Sensors

The S1 supports were prepared by immersing 24 NAA scaffolds (rectangular sheets were previously cut in small disks of 2 mm in diameter) in 8 mL of (3‐aminopropyl)triethoxysilane (0.22 mmol) solution in toluene for 6 h using an orbital stirrer at 50 rpm. In the next step, the functionalized supports were washed 3 times with 8 mL of toluene, 8 mL of acetone, and dried overnight at 60°C. Afterward, dried supports were stirred with 8 mL of RhoB solution in acetonitrile (1 mM) during 18 h to obtain S1 supports.

To prepare the final sensors S2, S1 supports were immersed in a capping solution of oligonucleotide O1 in hybridization buffer (20 mM TRIS‐HCl, 37.5 mM MgCl_2_, pH 7.5). An optimized capping solution of 5 µL O1 (100 µM) in a final volume of 250 µL hybridization buffer was placed under shaking at 37°C overnight for functionalization of two S1 supports. Finally, gated supports were washed 3 times with 1 mL of hybridization buffer to remove the excess of unbound O1 on support's surface.

### 2.4 Quantification of the Loaded Dye

To calculate the amount of the RhoB that can be loaded in the porous structure of NAA films, a pair of independent S2 supports was soaked in 1 mL of hybridization buffer. A control support was stirred at 37°C while the other one was stirred at 90°C for 60 min to force the pore opening and dye release. The fluorescence of the medium was measured at 575 nm (λ_exc_ = 555 nm) using a Synergy H1 microplate reader (BioTek, Winooski, Vermont, USA). The concentration of released RhoB was calculated using a calibration curve at different dye concentrations. The experiment was performed in triplicate.

### 2.5 Bacterial Culture and DNA Extraction

*V. vulnificus* strains representative of the main phylogenetic lineages are shown in Table 1. The strains *V. parahaemolyticus* CECT511^T^, *V. cholerae* CECT514^T^, and *V*. *harveyi* CECT525^T^ (CECT; Spanish Type Culture Collection; T, type strain) were included as negative controls to assess sensor specificity. All detection experiments with *V. vulnificus* were performed using strain CECT4999, whereas the remaining strains were used to confirm the specificity of the biosensor. The strains were routinely cultured in LB (Luria–Bertani medium) and on LB agar, both supplemented with 0.5% NaCl (LB‐1 and LB‐1 agar) at 28°C for 24 h and were kept frozen at −80°C in LB‐1 plus 20% glycerol. DNA was extracted from overnight bacteria LB‐1 cultures using the GenElute Bacterial Genomic DNA (Sigma, Spain) kit according to the manufacturer's instructions.

**Table 1:** *Vibrio vulnificus* strains used in the study and their characteristics.

Strain*	Source	Geographic origin/year	Phylogenetic** lineage/clade	pv.** *piscis*	Subtyping markers***	
					*pilF* PCR	*fpcrp* PCR
CECT 4999	Diseased eel	Spain, 1999	L2 – clade E	+	+	+
YJ016	Human blood	Taiwan, 1993	L1	−	+	−
CECT 5689	Diseased eel	Spain, 2002	L2 – clade A	+	−	+
12	Healthy tilapia	Israel, 2002	L3	+	+	+

* CECT: Colección Española de Cultivos Tipo (Spanish Type Culture Collection).

** Phylogenetic lineage, clade assignment, and pathovar (pv. *piscis*) were previously determined according to (Roig et al. 2018).

*** Subtyping based on the human virulence‐associated (*pilF*) and fish virulence‐associated (*fpcrp*) PCR markers was previously reported according to (Roig et al. 2010) and (Roig et al. 2022), respectively.

### 2.6 Detection Protocol

The release of RhoB to the solution in the presence of the target complementary DNA was determined to evaluate the capacity of the S2 sensors for *V. vulnificus* detection. Two independent S2 supports were submerged in 900 µL of hybridization buffer in a typical experiment. Then, 100 µL of the complementary DNA oligonucleotide (10 µM) and 100 µL of the hybridization buffer were added to each support, respectively. Mixtures were stirred at 37°C and aliquots of both solutions were taken at 15, 30, 45, and 60 min. The fluorescence of the solution was measured at 575 nm (λ_exc_ = 555 nm) to assess dye release. Fluorescence data were normalized with respect to the maximum fluorescence value. Three replicates were performed in the experiment.

Furthermore, suspensions of *V. vulnificus* live cells in phosphate buffer saline (PBS) were used to evaluate sensor response. Each S2 sensor was immersed in 900 µL of hybridization buffer plus either 100 µL of bacterial suspension (10^4^ CFU (Colony Forming Units) mL^−1^) or 100 µL of hybridization buffer (negative control). Aliquots of each solution were obtained at scheduled intervals while shaking at 37°C. In both assays, the amount of dye released was detected monitoring the fluorescence of the solutions (λ_exc_ = 555 nm, λ_em_ = 575 nm) using a plate reader.

### 2.7 Signal Amplification Assay

Two S2 supports were submerged in 990 µL of hybridization buffer. Additionally, genomic DNA from *V. vulnificus* (1.16 × 10^−3^ μg mL^−1^) was denatured at 95°C for 5 min, then cooled immediately in an ice bath (3 min). Ten microliters of denatured genomic DNA was then added to one support and 10 µL of water was added to the control one. Both supports were incubated for 60 min at 37°C with shaking. After incubation, aliquots (100 μL) were taken to measure the fluorescence. Released RhoB amount was quantified using a calibration curve at different dye concentrations performed at the same time. The quantity of DNA detected in the experiment was then directly associated with the amount of RhoB released.

### 2.8 Sensitivity and Selectivity Assessment

For sensitivity and selectivity assessment, suspensions of *V. vulnificus* live cells in phosphate buffer saline (PBS) were used. To calculate the limit of detection (LOD) of the gated sensor, 10 S2 sensors were immersed in 1 mL of 10‐fold bacterial dilutions (from 1 × 10⁰ to 1 × 10⁴ CFU mL⁻¹, including a negative control without bacteria) in hybridization buffer (20 mM TRIS‐HCl, 37.5 mM MgCl_2_, pH 7.5). After 60 min incubation at 37°C, released RhoB was detected monitoring the fluorescence of the solutions (λ_exc_ = 555 nm, λ_em_ = 575 nm) using a plate reader.

To evaluate sensor's selectivity, different S2 sensors were suspended in 1 mL of hybridization buffer with cell cultures from *V. vulnificus, V. cholerae, V. harveyi*, and *V. parahaemolyticus* and a combination of them at a final concentration of each bacterium of 10^3^ CFU mL^−1^. A control support was mixed with hybridization buffer without added cells, and the mixtures were stirred for 60 min at 37°C. Finally, solution fluorescence at 575 nm (λ_exc_ = 555 nm) was measured to determine the amount of rhodamine B delivered.

Minor variations in the maximum percentage of dye release between different experiments (e.g., sensitivity vs. selectivity assays) are attributed to the inherent batch‐to‐batch variability of the functionalized NAA supports, although the relative performance and detection reliability remain consistent.

### 2.9 Sensor Assessment With Natural Samples

To validate the potential utility of the sensor for both detection and diagnosis of *V. vulnificus*, we used different types of samples that simulate human and animal infection, including fish serum, fish mucus, and human serum, as well as environmental samples obtained from a natural lake and neighboring beaches (Albufera lake and Perelló beach) near the city of Valencia. These matrices were selected to represent the main routes of transmission and exposure in a One Health context.

Fish serum and fish mucus were obtained from farmed eels according to Marco‐Noales et al. (2001) and human serum was purchased from Sigma Aldrich (Spain). All samples (except human serum and part of the brackish water samples) were sterilized by filtration (0.22 m filters (Millipore)). The S2 sensors were immersed in 800 µL of hybridization buffer mixed with 100 µL of each sample and then a suspension of *V. vulnificus* (100 µL, 10^3^–10^5^ CFU mL^−1^) was added. A mixture of 800 μL of hybridization buffer with 200 μL of the sample without adding the bacteria was used as a negative control.

Unsterilized brackish water samples were processed for the presence of *V. vulnificus* using the selected sensor and conventional microbiological methods as a control. The S2 sensors were immersed in 850 µL of hybridization buffer and 150 µL of unsterilized brackish water was added. Another S2 sensor was immersed in 1000 µL of hybridization buffer as a secondary control in both types of analysis. The solutions containing the sensors were then shaken for 60 min at 37°C and fluorescence was measured at 575 nm with an excitation wavelength of 555 nm to determine the amount of RhoB released from the porous supports.

Microbiological controls of the unsterilized brackish water were performed according to Pérez‐Roig et al. (2022). Briefly, the procedure consists of enrichment in alkaline peptone water (pH 8.6) for 6–8 h followed by identification with a *V. vulnificus*‐specific PCR using as target the same gene as the one selected for the sensor, *vvhA*.

### 2.10 Statistical Analysis

Data were expressed as mean ± standard deviation (SD) of three independent replicates. Calibration curves were constructed using linear regression analysis. All statistical analyses were performed using GraphPad Prism (version 9.0).

## 3 Results and Discussion

### 3.1 Development and Characterization of the Sensing System

A two‐step process was used to obtain S1 support from NAA films. In the first step, the outer surface of NAA supports (S0) was functionalized with APTES in toluene and subsequently, supports were loaded with RhoB using a RhoB solution in acetonitrile. When we attempted to carry out dye loading and surface functionalization in the same reaction mixture in acetonitrile, a change of the optical properties of RhoB and uncontrolled hydrolysis of APTES were observed, that resulted in the formation of a solid phase on the NAA surface. This was avoided when the two‐step procedure described above was performed.

**Figure 1:**
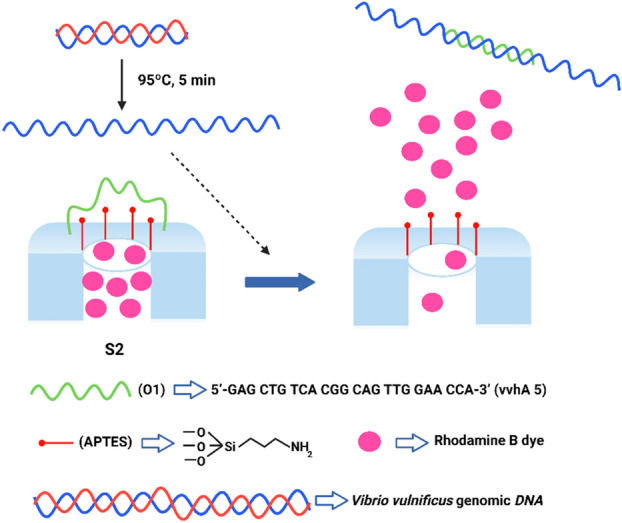
Schematic representation of the fluorogenic biosensor (S2) based on oligonucleotide‐gated nanoporous anodic alumina (NAA) films for *Vibrio vulnificus* detection. NAA films were loaded with rhodamine B (RhoB) and capped with an oligonucleotide probe containing a specific sequence of the *vvhA* cytolysin gene unique to *V. vulnificus*. In the presence of the target DNA, the probe is displaced, pores open, and the dye is selectively released into the medium.

In the next step, oligonucleotide O1, containing a partial base sequence of *V. vulnificus* cytolysin *vvhA* gene, was linked to the external surface through electrostatic interaction between the positively charged amino moieties on the support's surface and the negatively charged oligonucleotide backbone, resulting in the final sensor material S2. As shown in Figure 1, the oligonucleotide probe on the sensor surface is expected to block the pores preventing dye release while hybridization of the pathogen complementary ssDNA to the oligonucleotide probe on the sensor surface is expected to cause pore opening when *V. vulnificus* is present, allowing the release of the entrapped RhoB.

**Figure 2:**
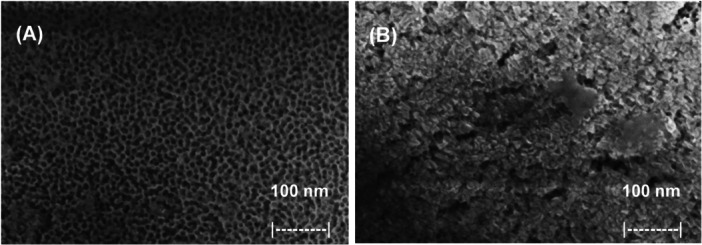
High‐resolution field emission scanning electron microscopy (HR‐FESEM) images of (A) the pristine NAA scaffold (S0) and (B) the final biosensor (S2) functionalized with the *vvhA* oligonucleotide probe. Images were acquired using a ZEISS GeminiSEM 500 at 5 kV. Scale bars = 100 nm. The porous structure of NAA is clearly visible in panel A, while the organic coating corresponding to the capping oligonucleotides is evident in panel B.

HR‐FESEM and EDXS analysis were performed on the initial NAA scaffold and on S1 and S2. As described by the provider, the NAA support (InRedox) is composed of anodic aluminum oxide films grown on 0.1 mm thick aluminum layers with a pore density 9 · 10^11 ^cm^−2^. In the upper part of the funnel, the pores are 20–30 nm in size that decrease to ca. 5 nm in the lower part. It was calculated that 4 ng of RhoB per g of NAA can be loaded and released with this pore morphology. HR‐FESEM images of the NAA scaffold (Figure 2A) and S2 sensor (Figure 2B) confirmed the porous structure of the NAA starting support, while the final S2 biosensor shows the outer surface coated with an organic phase corresponding to the capping oligonucleotides.

To determine the atomic content of the prepared materials (S0, S1, and S2), energy dispersive X‐ray spectroscopy (EDXS) was carried out (Table 2). The S1 support has high C/Al content after dye loading and surface functionalization with aminopropyl moieties, which decreased in S2 due to partial dye leakage during the O1 coating process and incomplete pore blocking of some of the pores in the material by O1. Additionally, the presence of aminopropylsilyl groups is supported by the presence of N and Si on S1 and S2 supports. Finally, P was present in the final material S2, indicating the presence of the O1 coating oligonucleotide, which was not detected in the NAA and S1 supports.

**Table 2:** Elemental ratios (C/Al, N/Al, Si/Al, and P/Al) in the different supports determined by energy dispersive X‐ray spectroscopy (EDXS).

Support	Elemental ratios*			
	C/Al	N/Al	Si/Al	P/Al
S0	0.12 ± 0.02	—	—	—
S1	1.98 ± 0.05	0.42 ± 0.01	0.15 ± 0.02	—
S2	0.67 ± 0.03	0.51 ± 0.02	0.11 ± 0.01	0.02 ± 0.01

* Data are expressed as mean ± standard deviation (*n* = 3 independent measurements). The increase in N and Si indicates surface functionalization, and the presence of P confirms attachment of the oligonucleotide probe in S2.

### 3.2 Dye Release Kinetics

**Figure 3:**
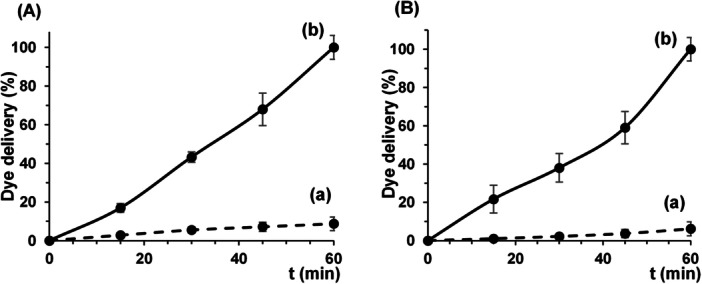
Rhodamine B release profiles from biosensor S2. (A) Delivery in the absence (black) and presence (red) of the complementary oligonucleotide O1c (1 µM) in hybridization buffer. (B) Release in the absence (black) and presence (red) of *V. vulnificus* cells (10³ CFU mL⁻¹) in hybridization buffer. Aliquots were taken at scheduled intervals during 60 min incubation at 37°C. Data are means ± standard deviation (*n* = 3).

The ability of the S2 biosensor to detect the *V. vulnificus* DNA region of interest as a function of time was first studied using an oligonucleotide complementary to the O1 oligonucleotide probe that covers the surface of the support. Figure 3A shows the release profile of RhoB from S2 in the presence and absence of the complementary oligonucleotide O1c. When the target oligonucleotide O1c was present, a remarkable release of dye from the pores was observed due to hybridization with the capping oligonucleotide O1 followed by pore opening. In contrast, when the complementary oligonucleotide was absent, a very low level of dye was released, indicating that the probes electrostatically attached to the scaffold surface effectively blocked the pores preventing dye delivery (Figure 1). This transduction mechanism differs substantially from classical “binding site‐signaling subunit” probes. Sensors based on gated materials perform their signaling by releasing a large number of trapped dyes independently of the stoichiometry of the host–guest interaction on their surface. This protocol allows the development of detection systems with high sensitivity due to signal amplification (vide infra).

In a second step, the S2 sensor was evaluated to detect *V. vulnificus* in a bacterial suspension as a function of time. RhoB release was negligible without bacteria, as shown in Figure 3B, suggesting that the O1 oligonucleotide anchored to the scaffold surface effectively closed the pores. In contrast, diffusion of the dye into the aqueous solution was remarkable when *V. vulnificus* cells were present (Figure 3B). Specific recognition of the bacterial DNA by the gated biosensor caused hybridization with the oligonucleotide probe on the surface of the support, displacement of the dsDNA, unblocking the pores and allowing the delivery of the dye. After these experiments, the time point of RhoB release at 60 min was fixed for the rest of studies due to the maximum difference of intensity between absence and presence of *V. vulnificus* DNA. Of note is the high performance of the sensor for direct detection of cellular DNA without sample pre‐treatment for DNA extraction and amplification. The incubation in hybridization buffer, which contains TRIS in saline medium, at 37°C may enhance cell permeation and the accessibility of the target DNA sequences, facilitating their interaction with the biosensor's gating sequences even in the absence of a formal extraction protocol, thereby facilitating direct detection.

### 3.3 Analytical Performance: Sensitivity and Specificity Studies

**Figure 4:**
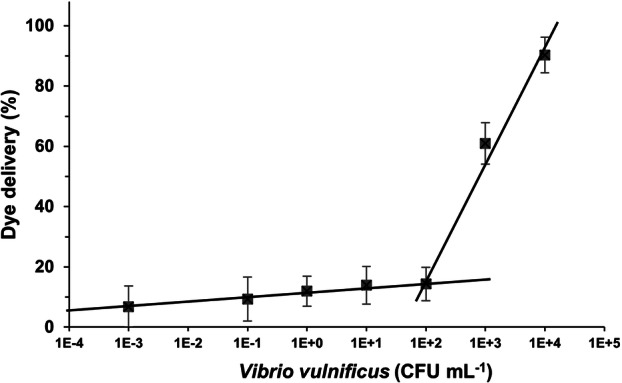
Analytical performance of the biosensor in terms of sensitivity. Rhodamine B release from S2 was measured after 60 min incubation at 37°C in 10‐fold serial dilutions of *V. vulnificus* (0–10⁴ CFU mL⁻¹). The limit of detection (LOD) was calculated as 10² CFU mL⁻¹ based on the intersection of the slopes of the response curve. Data represent mean ± standard deviation (*n* = 3).

The limit of detection (LOD) of the biosensor was calculated by determining the amount of dye released by S2 after 60 min of incubation at different cell concentrations in the range between 0 and 10^4^ CFU mL^−1^. A LOD of 10^2^ CFU mL^−1^ was calculated based on the intersection point of the two slopes of the plotted curve (Figure 4). These results showed that the dye delivered was directly related to the concentration of *V. vulnificus*, supporting the transduction mechanism detailed above. Furthermore, the LOD obtained is within the range of the previously reported most sensitive methods for the detection of *V. vulnificus*. Multiple studies using qPCR have reported LODs of 50–200 copies L⁻¹. Although direct conversion to molar concentration depends on genome size and reaction volume, these values broadly align with the sensitivity achieved here (Yang et al. 2020). Besides, our proposed method has a lower LOD than a DNAzyme‐based biosensor that detects *V. vulnificus* at about 10^3^ CFU mL^−1^ (Fan et al. 2021). According to the suggested detection mechanism, the amount of dye released depends on the amount of target DNA that would cause displacement of the coating oligonucleotide and pore opening. Recent studies have shown that the number of dye molecules released per DNA molecule in similar gated sensors is in the 10^4^–10^11^ range (Pla et al. 2021; López‐Palacios et al. 2025). To determine the amplification capacity of the biosensor, the S2 support was incubated with a known amount of denatured genomic DNA from *V. vulnificus* (1.16 × 10^−3^ μg mL^−1^). Released RhoB amount from porous support was quantified by fluorescence measurements using a calibration curve at different dye concentrations. The quantity of RhoB released from the sensor was then directly associated with the amount of DNA detected in the linear detection range (10^2^–10^4^ CFU mL^−1^). The amplification capacity of the gated porous sensor was determined resulting in 1.3 × 10^5^ molecules of RhoB released per genomic DNA molecule.

**Figure 5:**
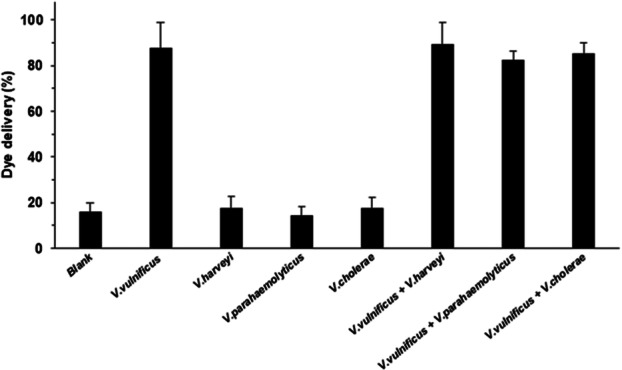
Specificity of biosensor S2 against different *Vibrio* species. Sensors were incubated for 60 min at 37°C in the presence of *V. vulnificus*, *V. harveyi*, *V. parahaemolyticus*, *V. cholerae* (10³ CFU mL⁻¹), or mixtures of *V. vulnificus* (10³ CFU mL⁻¹) with each of the other species (10³ CFU mL⁻¹). Dye release was only significant in the presence of *V. vulnificus*. Data are mean ± standard deviation (*n* = 3).

A second assay was conducted to determine the specificity of the biosensors by evaluating the response of S2 to different *Vibrio* species (*V. harveyi, V. parahaemolyticus*, and *V. cholerae*), as well as mixtures of them (*V. vulnificus* + *V. harveyi, V. vulnificus* + *V. parahaemolyticus*, and *V. vulnificus *+* V. cholerae*). Dye release from nine independent S2 supports was monitored at a value of 10^3^ CFU mL^−1^ of each *Vibrio* species in the medium after 60 min of incubation. The dye delivered from S2 in the presence of other *Vibrio* species was similar to the released RhoB amount from the blank assay and there was only a noticeable release of dye when *V. vulnificus* was present (Figure 5). Furthermore, similar fluorescence signal enhancement was observed when *V. vulnificus* was alone or in combination with other *Vibrio* species at 10³ CFU mL⁻¹ of each bacterium. These results demonstrate the ability of the S2 sensors to selectively detect *V. vulnificus* (Figure 5).

### 3.4 *V. vulnificus* Detection in Artificially Inoculated Natural Samples

**Figure 6:**
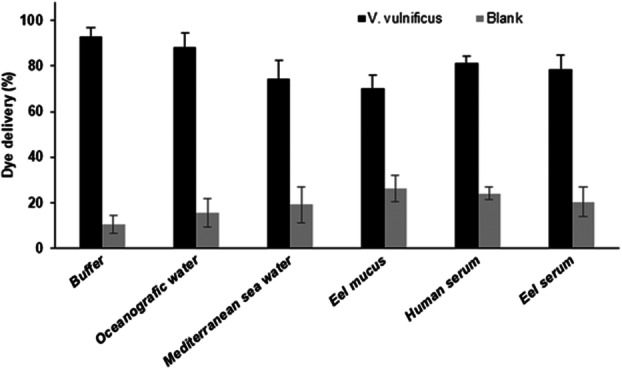
Performance of the biosensor in different matrices artificially inoculated with *V. vulnificus* (10³ CFU mL⁻¹). Samples included eel mucus, eel serum, human serum, and sterilized natural brackish water. In all cases, the sensor detected the pathogen through oligonucleotide displacement and dye release. Data are mean ± standard deviation (*n* = 3).

The robustness of the system was evaluated by determining the capacity of the S2 to detect *V. vulnificus* in different media simulating either environmental or clinical samples after 60 min of incubation. To this end, *V. vulnificus* cells were inoculated at 10^3^ CFU mL^−1^ into previously sterilized brackish water from natural ecosystems, fresh eel mucus, and human and eel serum. In all media, *V. vulnificus* caused a displacement of the gating oligonucleotide and dye delivery (Figure 6).

Finally, the LOD of S2 was also calculated in sterilized natural brackish water inoculated with different *V. vulnificus* cell concentrations (0–10^5^ CFU mL^−1^). The amount of dye released was proportional to the *V. vulnificus* cell concentration, resulting in a LOD of 500 CFU mL^−1^. Despite the absence of previous culture, sample treatment, or amplification steps, the LOD obtained with this probe is comparable to that previously found in buffer media (Figure 4) and to that found by Yang et al. (2020) using qPCR. This result demonstrates the potential of the sensor to analyze *V. vulnificus*‐containing samples quickly and efficiently. It was also demonstrated that the S2 sensors can be stored for weeks and used without any change in the sensing properties of supports, paving the way for potential in situ applications (Ribes et al. 2017).

### 3.5 *V. vulnificus* Detection in Natural Samples

Currently, the method of identification of *V. vulnificus* varies depending on whether the sample to be tested contains only *V. vulnificus* (clinical sample of vibriosis case) or not (environmental sample). In the former case, direct identification is feasible by any of the mentioned methods (PCR, qPCR, biosensors, etc.) while in the latter, *V. vulnificus* DNA must first be amplified by microbiological (enrichment in specific media) or genetic (different types of PCR) methods. In all these techniques, direct identification of *V. vulnificus* from environmental samples is not evaluated because *V. vulnificus* is outcompeted by other bacterial species (Liang et al. 2021; Lee et al. 2022; Bonny et al. 2022).

To find out whether our S2 biosensor would be able to detect *V. vulnificus* from natural waters, we performed a proof‐of‐concept evaluation by sampling in ecosystems near Valencia (Spain) (Table 3) and proceeded to the analysis and detection of this pathogen using S2 directly with the water samples and, in parallel, using the protocol reported by Pérez‐Roig et al. (2022). This protocol involves a prior enrichment in alkaline peptone water for 8 h followed by DNA isolation and PCR detection. The assays were performed in triplicate. As shown in Table 4, after 60 min of incubation, all samples in which *V. vulnificus* was detected directly using the S2 biosensor without a prior enrichment step were also positive using the reference method based on PCR detection after enrichment in broth. Therefore, in this proof‐of‐concept evaluation, the biosensor showed 100% agreement with the reference method. Significantly, our method offers several advantages over conventional reference methods, such as simplified sample handling since no DNA extraction or amplification is required. The assay provides reliable results in approximately 60 min, which is considerably faster than the 8‐h enrichment required for standard protocols. Furthermore, while this proof‐of‐concept study utilized a microplate reader, the system's design is amenable to miniaturization, potentially reducing dependence on expensive laboratory equipment and facilitating its future use by personnel with minimal training in field settings.

**Table 3:** Main physicochemical properties (temperature and conductivity) of water samples collected at different sites (Albufera lake and Perellonet beach, Valencia, Spain).

Sampling point	Water temperature (°C)	Water conductivity (μS/cm)*
Perellonet beach	18	1692
Natural park: Albufera lake	18	1627
Natural park: Albufera lake	9	1741
Perellonet beach	13	51,700
Perellonet beach	8	1765
Perellonet beach	8	1794
Perellonet beach	8	1821
Natural park: Albufera lake	8	1615
Natural park: Albufera lake	20	1606
Perellonet beach	19.5	4890
Perellonet beach	20.5	1680
Perellonet beach	20.5	1721
Natural park: Albufera lake	20	1947

* Conductivity values are proxies for salinity. pH was not determined in this study.

**Table 4:** Comparison of *V. vulnificus* detection in natural water samples using the biosensor (S2) and the reference method (enrichment + PCR) (Roig et al. 2022).

# Sample	Reference method (enrichment plus PCR detection)*	S2*
1	+	+
2	+	+
3	−	−
4	−	−
5	−	−
6	−	−
7	−	−
8	−	−
9	+	+
10	+	+
11	+	+
12	+	+
13	+	+

* Samples were analyzed in triplicate. Biosensor results were considered positive when fluorescence intensity exceeded three standard deviations of negative controls at 575 nm (λ_exc_ = 555 nm).

## 4 Conclusions

In summary, we have developed and rigorously tested a selective and sensitive fluorogenic biosensor for detecting *V. vulnificus*, utilizing nanoporous materials combined with short DNA sequences tailored specifically for this purpose. The biosensor is based on the use of NAA scaffolds loaded with RhoB and coated with an oligonucleotide targeting a sequence unique to *V. vulnificus*. The interaction with the target bacteria DNA triggers the opening of the pores and the subsequent release of RhoB, allowing for the detection of the pathogen. This biosensor demonstrated exceptional selectivity for *V. vulnificus*, showing no cross‐reactivity with other *Vibrio* species, and exhibited limits of detection (LOD = 10^2^ CFU mL^−1^) in natural water samples comparable to those achieved by other state‐of‐the‐art *V. vulnificus* detection methods. Its effectiveness was proven in detecting *V. vulnificus* directly from simulated‐natural and ‐clinical samples, as well as natural environmental samples, demonstrating its potential for monitoring coastal water quality and for the rapid diagnosis of vibriosis in humans and animals. Furthermore, by employing various reporters and capping sequences, this sensor design could be easily adapted for the multiplexed detection of the four most clinically significant *Vibrio* species, *V. vulnificus*, *V. parahaemolyticus*, *V. alginolyticus*, and *V. cholerae*, highlighting its versatility and broad applicability in public health and environmental monitoring. This work demonstrates the applicability of the biosensor across clinically and environmentally relevant matrices, supporting its potential for both diagnostic and monitoring purposes. Nevertheless, these results should be interpreted as proof‐of‐concept rather than a full validation, and to enable eventual field validation and routine implementation.

## Author Contributions

**M. Nieves Aranda:** investigation, methodology, writing – original draft. **Isabel Caballos:** investigation and methodology. **Alba López‐Palacios:** investigation and methodology. **Héctor Carmona‐Salido:** investigation and methodology. **Eva Sanjuan:** investigation and methodology. **Elena Aznar:** conceptualization, funding acquisition, writing – review and editing, supervision. **Carmen Amaro:** conceptualization, funding acquisition, writing – review and editing, supervision. **Ramón Martínez‐Máñez:** conceptualization, funding acquisition, writing – review and editing, supervision. **Andy Hernández‐Montoto:** conceptualization, writing – review and editing, supervision, validation.

## Ethics Statement

The authors have nothing to report.

## Consent

The authors have nothing to report.

## Conflicts of Interest

The authors declare no conflicts of interest.

## Data Availability

The data that support the findings of this study are available from the corresponding author upon reasonable request.
